# Treatment of Small Ruptured Intracranial Aneurysms: Comparison of Surgical and Endovascular Options

**DOI:** 10.1161/JAHA.112.002865

**Published:** 2012-08-24

**Authors:** Nohra Chalouhi, David L. Penn, Stavropoula Tjoumakaris, Pascal Jabbour, L. Fernando Gonzalez, Robert M. Starke, Muhammad S. Ali, Robert Rosenwasser, Aaron S. Dumont

**Affiliations:** Department of Neurological Surgery, Thomas Jefferson University and Jefferson Hospital for Neuroscience, Philadelphia, PA

**Keywords:** aneurysm, small, aneurysm, ruptured, clipping, endovascular procedures

## Abstract

**Background:**

Small intracranial aneurysms pose significant challenges to endovascular therapy. Surgical clipping is considered by many to be the preferred treatment for these lesions. We present the results of the first study comparing the 2 treatment modalities in small ruptured aneurysms.

**Methods and Results:**

Between 2004 and 2011, 151 patients with small ruptured aneurysms (≤3 mm) were treated in our institution: 91 (60.3%) with endovascular therapy and 60 (39.7%) with surgical clipping. The surgical and endovascular groups were generally comparable with regard to baseline demographics, with the exception of larger mean aneurysm size in the endovascular group versus the surgical group (2.8 versus 2.5 mm, respectively; *P*<0.001) and a higher proportion of posterior circulation aneurysms in the endovascular group. Endovascular treatment failed in 9.9% of patients. Procedure-related complications occurred in 23.3% of surgical patients versus 9.8% of endovascular patients (*P*=0.01). Only 3.7% of patients undergoing endovascular therapy experienced an intraprocedural aneurysm rupture. There were no procedural deaths or rehemorrhages in either group. The rates of aneurysm recanalization and retreatment after endovascular therapy were 18.2% and 12.7%, respectively. Favorable outcomes (moderate, mild, or no disability) were not statistically different between the endovascular (67.1%) and surgical (56.7%) groups (*P*=0.3).

**Conclusions:**

Surgical clipping was associated with a higher rate of periprocedural complications, but overall disability outcomes were similar. Endovascular therapy, if technically feasible, might be a preferred option in this setting. Inclusion of patients with small aneurysms in randomized controlled trials seems feasible and will be needed to provide definitive information on the best therapeutic approach. **(*J Am Heart Assoc*. 2012;1:e002865 doi: 10.1161/JAHA.112.002865.)**

## Introduction

Endovascular therapy has become the preferred treatment modality for ruptured aneurysms in many centers since the publication of the International Subarachnoid Aneurysm Trial (ISAT), which showed an outcome benefit with endovascular coiling compared to surgical clipping.^1–2^ However, the investigators of the ISAT excluded small aneurysms (≤3 mm) from the trial, given the technical challenges that they pose for endovascular therapy, thus leaving questions about the best management strategy for patients with small aneurysms unanswered. Difficulties in catheterizing the aneurysm, stabilizing the microcatheter, and deploying coils into confined spaces have raised questions about the feasibility and safety of endovascular therapy in small aneurysms. As such, many investigators have reported a high risk of intraprocedural rupture with coiling of very small aneurysms. Nguyen et al^3^ demonstrated that patients undergoing endovascular coiling of small aneurysms were 5 times more likely to experience intraprocedural ruptures than were patients with larger aneurysms. Furthermore, the risk is more than twice as high in ruptured (10.7%) compared to unruptured aneurysms (5.0%), according to a recent meta-analysis that included 422 small aneurysms (≤3 mm) treated with endovascular coiling.^4^ Despite this potential drawback, endovascular treatment of small aneurysms is feasible and effective according to multiple recent reports.^4–7^ Nevertheless, given the proven role of microsurgery in small aneurysms and the perceived challenges with endovascular therapy, surgical clipping is considered by many to be the preferred treatment modality in this setting. Currently, there are no studies comparing neurosurgical clipping with endovascular coiling in patients with small ruptured aneurysms (SRA).

In the present study, the authors assess their experience with surgical and endovascular treatment of SRA and compare the 2 treatment modalities in terms of safety, feasibility, and patient outcome.

## Methods

The study protocol was approved by the Thomas Jefferson University Institutional Review Board. We searched our prospectively maintained database for all patients with SRA undergoing endovascular coiling or surgical clipping between 2004 and 2011 at our institution. Only patients with aneurysms ≤3 mm in their greatest dimension, as measured by 2D or 3D digital subtraction angiography (DSA), were included in the study. This was based on the adopted definition for small aneurysms in most series.^4–7^ A total of 151 patients with SRA were identified. There were 91 patients in the endovascular group and 60 in the neurosurgical group. Medical charts, angiographic studies, magnetic resonance imaging (MRI) scans, and computed tomographic (CT) scans were carefully reviewed. Patients' age, sex, Hunt and Hess grades, and aneurysm locations were recorded. For endovascular coiling, the degree of aneurysm occlusion was determined by the operator at the time of the procedure. Any aneurysm that displayed a decreasing percentage of occlusion on follow-up angiography was considered recurrent. Thromboembolic and ischemic complications were diagnosed clinically (new deficits or change in level of consciousness) or on CT/MRI scans (new infarcts) after exclusion of confounders like vasospasm, hydrocephalus, and metabolic disorders. CT/MRI studies typically were performed in cases of sudden neurological compromise. Thromboembolic complications also were diagnosed intraoperatively on DSA for endovascular procedures. Intraprocedural aneurysm ruptures occurring during endovascular coiling were recorded, along with the associated morbidity. Other procedural complications in the surgical group, including epidural/subdural hematomas, infections, and postoperative seizures, were reported as well. The presence of vasospasm was assessed by the development of a focal neurological deficit or change in level of consciousness with confirmatory transcranial Doppler, CT angiography, or DSA, as necessary, and other causes were excluded. Clinical outcome was evaluated at time of discharge with the Glasgow Outcome Scale (GOS). GOS was determined on the basis of a patient's neurological examination and functional status at discharge and was classified as follows: I, deceased; II, vegetative state; III, severely disabled; IV, moderately disabled; and V, mildly disabled or not disabled. Angiographic follow-up (DSA or magnetic resonance angiography [MRA]) was scheduled at 6 months, 1 year, 2 years, and 5 years after endovascular procedures. We routinely perform intraoperative angiography in all patients undergoing neurosurgical clipping.

All procedures were performed by neurosurgeons trained in both microsurgical clipping and endovascular embolization. In most cases, patients were eligible for both treatment modalities. Factors like patient characteristics, aneurysm features, and operator's preferences were taken into account in the decision-making process. Patients with posterior circulation aneurysms were offered primarily endovascular therapy. Conversely, patients with a large intraparenchymal hematoma underwent open surgery for aneurysm clipping and simultaneous clot evacuation and decompression. Elderly patients with multiple comorbidities or poor neurological grades often were preferentially offered an endovascular procedure. Aneurysms with wide necks or unfavorable neck-to-dome ratio were either clipped or coiled with stent or balloon assistance.

### Endovascular Treatment

**Figure 1.**
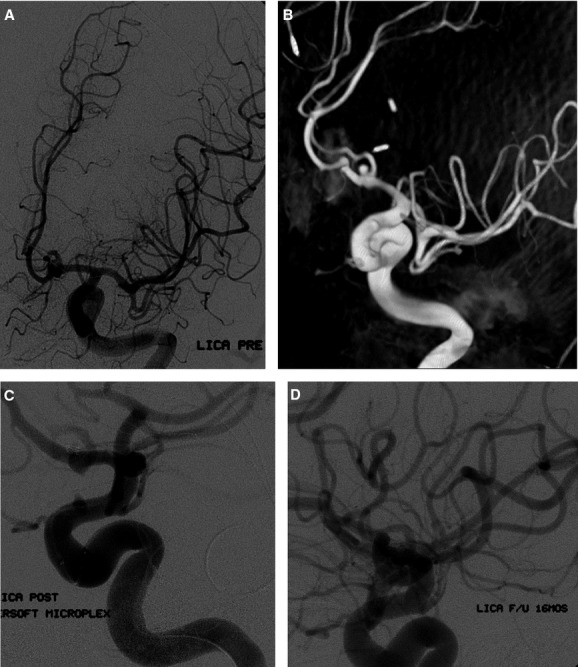
Frontal view (A) and 3D reconstruction (B) of DSA showing a left-sided 2.3×1.5-mm anterior communicating artery aneurysm in a patient with a Hunt and Hess Grade III subarachnoid hemorrhage. The aneurysm was successfully embolized with coils. Lateral views of initial (C) and follow-up (16 months) angiograms (D) showing complete obliteration of the aneurysm.

**Figure 2.**
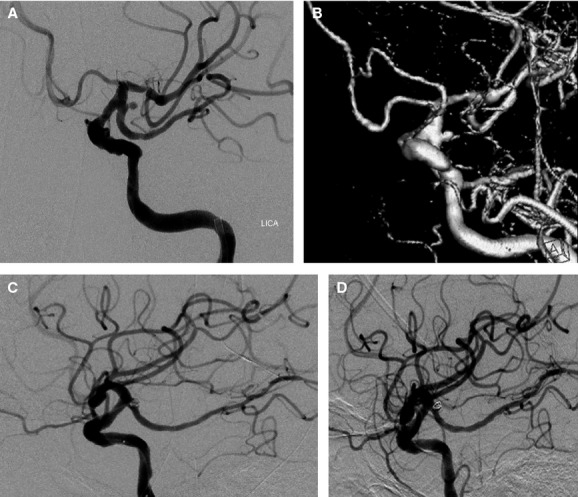
Lateral view (A) and 3D reconstruction (B) of DSA showing a 2.9×2.0-mm aneurysm arising from the left posterior communicating artery in a patient with a Hunt and Hess Grade IV subarachnoid hemorrhage. The aneurysm was successfully coiled (C) and maintained adequate occlusion at the 1-year follow-up (D).

All patients underwent arterial line and central venous line placement preoperatively. Patients with Hunt and Hess Grade III or higher also were monitored with ventriculostomy and Swan-Ganz catheters. Endovascular procedures were performed under general anesthesia and continuous neurophysiological monitoring, including somatosensory-evoked potentials, brainstem auditory-evoked responses, and electroencephalography. An initial bolus of 50 U/kg heparin generally was administered after deployment of the first coil, and activated clotting time was maintained intraoperatively at 2 times the patient's baseline. For stent-assisted procedures performed in the setting of subarachnoid hemorrhage, patients also were loaded with 600 mg clopidogrel intraprocedurally. Coils were placed until satisfactory aneurysm obliteration was achieved or placement of additional coils was not possible (Figures 1 and 2). Depending on operator preferences, wide-necked aneurysms were coiled either with stent assistance (with Neuroform [Stryker Neurovascular, Fremont, CA] and Enterprise [Cordis Neurovascular, Miami, FL] stents) or with balloon assistance (with the Hyperglide balloon [eV3, Irvine, CA]). The procedure was aborted if coiling was deemed to be technically challenging or hazardous or if an attempt to deploy coils failed. The patient then was transferred immediately to the operating room for microsurgical clipping. When an intraprocedural aneurysm rupture occurred, heparin (if it had been administered) was reversed with protamine, and coils were deployed rapidly to secure the aneurysm.

### Surgical Clipping

**Figure 3.**
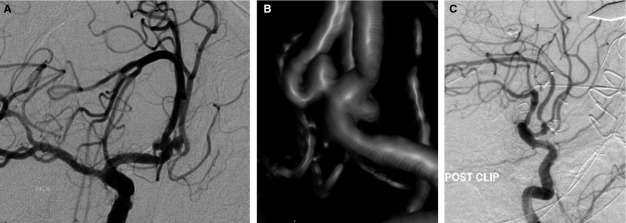
Frontal view (A) and 3D reconstruction (B) of preoperative DSA demonstrating a 2.5×2.6-mm wide-necked aneurysm arising from the anterior communicating artery in a patient with a Hunt and Hess Grade IV subarachnoid hemorrhage. Intraoperative angiography (C) was performed after clip placement and demonstrated complete obliteration of the aneurysm with patency of afferent and efferent parent vessels.

Microsurgical aneurysm treatment was performed immediately after diagnostic angiography. Procedures were performed under general endotracheal anesthesia and with administration of corticosteroids and diuretics. Continuous neurophysiological monitoring, including somatosensory-evoked potentials, brainstem auditory-evoked responses, and electroencephalography, was performed in all cases; motor-evoked potentials were monitored at the discretion of the primary surgeon. Different approaches were used according to the location of the aneurysm. Patients were placed in burst suppression (6 to 8 bursts/minute) if temporary clipping was used. Intraoperative angiography was performed in all cases to document aneurysm obliteration, patency of feeding vessels, and the need for clip readjustment (Figure 3).

### Postoperative Care

Patients were monitored closely in the neuro-intensive care unit after aneurysm treatment. Prophylactic nimodipine was administered routinely to prevent the development of cerebral vasospasm. Patients with hydrocephalus were managed with ventriculostomy. A ventriculoperitoneal shunt later was inserted in patients who failed multiple attempts to wean from the ventriculostomy. Once patients had become neurologically and medically stable, they were discharged to home, to a rehabilitation facility, or to a skilled nursing facility according to their functional disabilities.

### Statistical Analysis

A comparative analysis between the surgical and the endovascular groups was conducted. Data are presented as mean and range for continuous variables and as frequency for categorical variables. Statistical analysis of categorical variables was carried out with χ^2^ and Fisher exact tests as appropriate. Comparison of means was carried out with the Student *t* test. Interaction and confounding were assessed through stratification and relevant expansion covariates. A multivariable logistic regression analysis was carried out to determine predictors of perioperative complications and patient outcome. Analysis of outcome was based on actual treatment (ie, patients crossing over from coiling to clipping were assigned to the clipping group). Factors predictive in univariable analysis (*P*<0.15)^8^ were entered into a stepwise backward multivariable logistic regression analysis. *P* values ≤0.05 were considered statistically significant. Statistical analysis was carried out in Stata 10.0 (StataCorp, College Station, TX).

## Results

### Baseline Characteristics

Of 151 patients with SRA included in the study, 91 (60.3%) underwent endovascular therapy and 60 (39.7%) underwent surgical clipping (Table 1). Mean age was similar in endovascular patients (53.9±15.8 years) and in surgical patients (51.8±12.9 years) (*P*=0.4). Mean aneurysm size was significantly higher in the endovascular group (2.8±0.3 mm) than in the surgical group (2.5±0.3 mm) (*P*<0.001). There was a higher proportion of patients with good Hunt and Hess grades (I and II) in the surgical group (43.3%) than in the endovascular group (29.7%), but the difference did not reach statistical significance (*P*=0.2). Hunt and Hess grades are summarized in Table 2. Aneurysms arising from the anterior communicating artery (67/151, 44.4%) and the posterior communicating artery (27/151, 17.9%) accounted for the majority of aneurysms (62.3%) in the study (Table 3). All posterior circulation aneurysms were treated by endovascular therapy (*P*=0.001).

**Table 1.** Demographics and Aneurysm Characteristics

Characteristics	Endovascular Therapy	Surgical Clipping	*P*
Total patients, n	91	60	
Mean age, y, mean±SD	53.9±15.8	51.8±12.9	0.4
Sex, female, n (%)	65 (71.4)	41 (68.3)	0.7
Aneurysm size, mm, mean±SD	2.8±0.3	2.5±0.3	<0.001*
Hunt and Hess Grades I–II, n (%)	27 (29.7)	26 (43.3)	0.2
Mean Hunt and Hess grade, mean±SD	2.7±1.1	2.5±1.2	0.3
Posterior circulation aneurysms, n (%)	14 (15.4)	0	0.001*
Anterior and posterior communicating artery aneurysms, n (%)	52 (57.1)	42 (70)	0.1

* Statistically significant values. SD indicates standard deviation.

**Table 2.** Hunt and Hess Grades

Hunt and Hess Grades	Endovascular Therapy, n (%)	Surgical Clipping, n (%)	Total, n (%)
I	21 (23.1)	19 (31.7)	40 (26.5)
II	6 (6.6)	7 (11.6)	13 (8.6)
III	41 (45.1)	19 (31.7)	60 (39.7)
IV	22 (24.1)	13 (21.7)	35 (23.2)
V	1 (1.1)	2 (3.3)	3 (2.0)
Total	91 (100)	60 (100)	151 (100)

**Table 3.** Location of SRA

Location	Endovascular Therapy, n (%)	Surgery, n (%)	Total, n (%)
Anterior communicating	35 (38.5)	32 (53.3)	67 (44.4)
Posterior communicating	17 (18.6)	10 (16.7)	27 (17.9)
Vertebral	9 (9.9)	0	9 (5.9)
Basilar	5 (5.5)	0	5 (3.3)
Pericallosal	8 (8.8)	4 (6.7)	12 (8.0)
Middle cerebral	7 (7.7)	6 (10)	13 (8.6)
Carotid terminus	6 (6.6)	5 (8.3)	11 (7.2)
Posterior carotid wall	2 (2.2)	1 (1.7)	3 (2.0)
Anterior choroidal	1 (1.1)	2 (3.3)	3 (2.0)
Superior hypophyseal	1 (1.1)	0	1 (0.7)
Total	91 (100)	60 (100)	151 (100)

SRA, small ruptured aneurysms.

### Aneurysm Treatment

Of 91 patients in the endovascular group, 81 were treated with conventional coiling, 5 with stent-assisted coiling, 4 with balloon-assisted coiling, and 1 with Onyx HD 500 (eV3). Nine (9.9%) of 91 patients had unsuccessful endovascular procedures. Surgical clipping was performed in 7 of these patients, and care was withdrawn in 2 other patients because of poor neurological grades. Of 82 patients undergoing successful endovascular treatment, 79 (96.3%) had complete or near-complete aneurysm occlusion (>95%) at the end of the procedure. There were 8 (9.8%) overall procedure-related complications in this group: Three (3.7%) thromboembolic or ischemic events (including 2 clinically silent infarcts), 3 (3.7%) intraprocedural ruptures, and 2 (2.4%) retroperitoneal hematomas not requiring any intervention. Procedural complications led to permanent morbidity in 3 (3.7%) patients (2 intraprocedural ruptures and 1 ischemic event). Two of these 8 procedural complications occurred in stented patients (1 clinically silent infarct and 1 retroperitoneal hematoma). The rate of procedural complications for patients treated with conventional coiling (excluding stent and Onyx embolization) was 7.9% (6/76). No procedure-related deaths occurred in the endovascular group.

Aneurysm clipping was successful in all 60 patients included in the surgical group. Intraoperative angiography showed complete obliteration of all clipped aneurysms. Procedure-related complications were seen in 14 (23.3%) patients. There were 7 (11.7%) ischemic events (including 3 clinically silent infarcts), 5 (8.3%) postoperative epidural (4/5) or subdural (1/5) hematomas requiring emergent evacuation, 1 (1.7%) subgaleal infection, and 1 (1.7%) acute postoperative seizure. Procedural complications led to permanent morbidity in 5 (8.3%) patients (4 ischemic events and 1 subdural hematoma). No procedure-related deaths occurred in this group. No patient crossed over from the surgical group to the endovascular group.

The difference in complication rates between surgical and endovascular treatment (23.3% versus 9.8%, respectively) was found to be statistically significant (*P*=0.01), but the difference in procedure-related permanent morbidity rates (8.3% versus 3.7%) fell short of statistical significance (*P*=0.2). In univariable analysis, surgical treatment (*P*=0.01) and Hunt and Hess Grades III to V (*P*=0.09) were associated with perioperative complications. In multivariable logistic regression analysis, surgical treatment was the only predictor of perioperative complications (odds ratio=2.4; 95% confidence interval, 1.2–8.4; *P*=0.03).

### Outcome

There was no significant difference in cerebral vasospasm development in the endovascular group versus the surgical group (24.4% [20/82] of endovascular patients and 35.0% [21/60] of surgical patients developed vasospasm [*P*=0.2]). Hydrocephalus developed in 57.3% (47/82) and 48.3% (29/60) of patients in the endovascular and surgical groups, respectively (*P*=0.3). Placement of a ventriculo-peritoneal shunt was required in 31.7% (26/82) of patients in the endovascular group and 28.3% (17/60) in the surgical group. At the time of discharge, a higher proportion of patients attained a favorable outcome (moderate, mild, or no disability) in the endovascular group (67.1%, 55/82) than in the surgical group (56.7%, 34/60), but the difference did not reach statistical significance (*P*=0.3) (Table 4). Three of the 7 patients who crossed over from coiling to clipping treatment had a favorable outcome (moderate, mild, or no disability). No patient experienced a rehemorrhage after endovascular or surgical treatment in this series.

**Table 4.** Clinical Outcomes of Patients With SRA

GOS	Endovascular Therapy, n (%)	Surgery, n (%)	Total, n (%)
I (death)	7 (8.5)	3 (5.0)	10 (7.0)
II (vegetative state)	2 (2.4)	2 (3.3)	4 (2.8)
III (severe disability)	18 (22.0)	21 (35.0)	39 (27.5)
IV (moderate disability)	30 (36.6)	18 (30.0)	48 (33.8)
V (mild/no disability)	25 (30.5)	16 (26.7)	41 (28.9)
Total	82 (100)	60 (100)	142 (100)

SRA, small ruptured aneurysms.

In univariable analysis, factors predicting a poor clinical outcome (GOS I to III versus GOS IV to V) were Hunt and Hess Grades III to V (*P*<0.001), posterior circulation aneurysms (*P*=0.05), and vasospasm (*P*=0.008). In multivariable logistic regression analysis, Hunt and Hess Grades III to V (odds ratio=0.08; 95% confidence interval, 0.03–0.2; *P*<0.001) were the only significant predictor of poor clinical outcome. Surgical treatment was not a predictor of poor outcome even after control for all other variables (odds ratio=0.6; 95% confidence interval, 0.3–1.4; *P*=0.2).

With the exclusion of 10 patients who died in the hospital or soon after discharge, angiographic follow-up was available in 55 (76.4%) of 72 patients (52 DSA, 3 MRA) treated with endovascular therapy at a mean time point of 9.5 months. The majority of those who missed their scheduled follow-up were patients who remained severely disabled after treatment. Aneurysm recanalization was seen in 10 (18.2%) of 55 patients at the last available follow-up. Seven (12.7%) of these patients had to be retreated: 5 with additional coiling and 2 with surgical clipping.

## Discussion

Small aneurysms ≤3 mm in diameter account for almost 6% of all ruptured intracranial aneurysms^9^ and pose a therapeutic dilemma for the treating physician. Published studies have investigated the safety and efficacy of endovascular therapy unilaterally without comparison to a surgical group, which limits the value of their findings.^3–5^ The present study is the first study to directly compare surgical clipping and endovascular therapy in a consecutive series of patients harboring SRA. We found that endovascular therapy has a significantly lower complication rate (>2-fold) than open surgery, with a low incidence of intraprocedural rupture (3.7%).

Although the difference did not reach statistical significance, patients undergoing endovascular therapy achieved a better clinical outcome than surgical patients despite a potential inherent selection bias (ie, patients with poor neurological grades or posterior circulation aneurysms were assigned more frequently to the endovascular group). In fact, the analysis in GOS subgroups probably was underpowered to detect small differences in patient outcome. Our findings add to the results of the ISAT^1^ and the recent Barrow Ruptured Aneurysm Trial (BRAT)^10^ and suggest that endovascular therapy might also be the preferred treatment modality for SRA. The ISAT reported a relative risk reduction of 22.6% in death or dependency with endovascular therapy as compared to open surgery.^1^ However, all patients with small aneurysms were excluded from ISAT. The BRAT, another landmark study, reported poor outcome at 1 year in 33.7% of patients treated with open surgery versus 23.2% of those who underwent endovascular treatment.^10^ The authors of BRAT did not specify how many small aneurysms were included, how they were treated, or what the outcome was in such patients. Furthermore, one of the main reasons for the high crossover rate from coiling to surgery in the BRAT (often thought to be a main weakness of this trial) was that small aneurysms were considered to be better suited for surgical clipping. In fact, our study and others suggest that even very small aneurysms can be treated effectively with endovascular therapy.^4–6^

The advent of adjunctive techniques, such as balloon- or stent-assisted coiling, and the introduction of soft and smaller coils (1.5 mm) have improved substantially the ability to treat small aneurysms by endovascular means. We found endovascular therapy to be feasible, safe, and effective in the treatment of SRA. The results of our study are in concert with emerging data suggesting that endovascular therapy can be performed safely in small aneurysms. Ioannidis et al^11^ reviewed their experience with endovascular therapy in 97 patients presenting with SRA and reported procedural complications in 7.2%, intraprocedural ruptures in 4.1%, and favorable outcomes in 80.3% of patients. In the meta-analysis by Brinjikji et al,^4^ the overall rate of morbidity for endovascular therapy in SRA was 4%, in line with the 3.7% in the present report. In contrast to many recently published studies, however, the rate of intraprocedural rupture with endovascular therapy was relatively low in our hands (3.7%). This potentially devastating complication has been regarded as the primary limitation of endovascular therapy in small aneurysms.^4^ The rate of intraprocedural ruptures during endovascular treatment of SRA was found to be as high as 10.1% by van Rooij et al^5^ and 16.7% by Brinjikji et al.^4^

Endovascular procedures failed in up to 10% of all cases in the present study, with a significant proportion of patients crossing over to surgical clipping. This high rate of failure with endovascular therapy underlines the technical challenges posed by small aneurysms and reflects our low threshold for aborting an endovascular procedure in favor of surgical clipping to prevent an intraprocedural aneurysm rupture. Aneurysm recanalization seems to be another limitation of endovascular therapy in SRA. In the present study, the rates of recurrence (18.2%) and retreatment (12.7%) were unexpectedly high given that only small aneurysms were included. We believe that the high recurrence rate reflects the greater propensity for ruptured aneurysms to recur^12–13^ as well as the difficulties of coil deployment into small confined spaces, with resultant aneurysm underpacking. According to the meta-analysis by Brinjikji et al,^4^ only 5.4% of small aneurysms require retreatment through either surgical clipping or additional coiling. However, in that meta-analysis, almost 40% of aneurysms were unruptured, which explains the apparent discrepancy with our results.

Surgical clipping of SRA was associated with a 23.3% complication rate in our study. However, permanent morbidity and mortality rates were limited to 8.3% and 0%, respectively. Surprisingly, epidural and subdural hematomas occurred in 8.3% of patients after surgery. A sampling error is the only plausible explanation we can provide for this finding. Although all patients in our study had aneurysms ≤3 mm in largest diameter, aneurysm size was still significantly smaller in surgical (2.5 mm) than in endovascular (2.8 mm) patients. This interesting finding underscores the fact that the smallest aneurysms continue to be an endovascular challenge, with surgical clipping being the only available option in such cases. Overall, despite having higher morbidity rates than endovascular therapy, surgical clipping remains a highly effective treatment option for SRA.

The limitations of our study are mainly related to its retrospective design, the relatively small sample size (with resulting low statistical power), and the absence of randomization. As discussed above, aneurysm size, location, and neurological condition (Hunt and Hess grade) differed between the 2 groups. Assessment of outcome at discharge is another limitation of our study because outcome at discharge might not reflect ultimate patient outcome. Additionally, because CT/MRI studies were not obtained routinely after clipping and coiling procedures, some clinically silent infarcts probably were missed.

## Conclusion

Lower procedural complication rates were seen with endovascular therapy as compared to open surgery in SRA. Overall patient outcomes, however, were similar in both groups. In light of our results, endovascular treatment of SRA seems to be feasible, safe, and effective and might be a preferred option in this setting. These data provide impetus for further study, in which SRA need not be excluded from comparisons of coiling and neurosurgical clipping. More specifically, the stage is set for a randomized controlled trial to compare the 2 treatment modalities in patients with SRA.
